# Status Competition and Implicit Coordination: Based on the Role of Knowledge Sharing and Psychological Safety

**DOI:** 10.3389/fpsyg.2022.871426

**Published:** 2022-05-02

**Authors:** Jiuling Xiao, Yushan Xue, Yichen Peng, Jiankang Wang

**Affiliations:** ^1^School of Business, Nanjing Audit University, Nanjing, China; ^2^School of Business Administration, Nanjing University of Finance & Economics, Nanjing, China; ^3^School of Public Administration, Nanjing Audit University, Nanjing, China

**Keywords:** implicit coordination, dominant-type status competition, prestige-type status competition, knowledge sharing, psychological safety

## Abstract

Implicit coordination is an important research topic in the field of social cognition. Previous studies have studied implicit coordination behavior from the perspective of team mental model but ignored the internal mechanism of individual status competition motivation on implicit coordination behavior. Based on the differences of status competition motivation, the individual status competition motivation is divided into prestige-type and dominant-type. With knowledge sharing as the mediating variable and psychological safety as the moderating variable, this research constructed a process model of the influence of status competition motivation on implicit coordination behavior. The empirical study was carried out with a sample of 367 employees of 44 enterprises. The research results show the following findings: (1) Status competition has a differentiated impact on implicit coordination. Prestige-type status competition has a significant positive impact on implicit coordination behavior, while dominant-type status competition has a significant negative impact on implicit coordination behavior. (2) Knowledge sharing plays a mediating role between status competition (prestige-type status competition and dominant-type status competition) and implicit coordination. (3) Psychological safety positively moderates the relationship between prestige-type status competition, dominant-type status competition, and knowledge sharing. The research results provide a new perspective for the field of implicit coordination; reveal the mechanism of status competition motivation in implicit coordination, which is of great significance to the practice of enterprise team management and human resource management.

## Introduction

Many enterprise coordination practices show that in a complex, dynamic, and uncertain environment, when employees are faced with high pressure and high load tasks, they will often adjust the coordination mode. Employees tend to change the explicit coordination to the implicit coordination, to reduce the process losses, improve coordination efficiency, and achieve task objectives (Stout et al., 1999; Colman and Gold, 2018; Stefanini et al., 2020). According to the theory of organizational coordination, coordination is the act of working together harmoniously by the employees, and the act of managing interdependencies between activities performed to achieve a goal (Malone and Crowston, 1992). According to the different coordination modes, it can be divided into explicit coordination and implicit coordination (Rico et al., 2008; Chang et al., 2017). Explicit coordination refers to visible and external coordination patterns under regulations or through interventions by administrators, including mutual communication, direct monitoring, standard operation procedures, or behavioral regulation plans, rules, and objectives (Blickensderfer et al., 2010; Fisher et al., 2012). On the contrary, implicit coordination refers to adjustment behavior driven by deep cognition, that is, the adjustment by team members of their behavioral model according to their anticipated tasks and other team members’ needs (Chang et al., 2017; Rico et al., 2019).

Implicit coordination and explicit coordination are two very important forms of coordination in an enterprise. Explicit coordination mainly focuses on the use of task organization mechanisms, such as work plans, meetings, and operation manuals, as well as communication mechanisms, such as oral, written, formal, and informal (Espinosa et al., 2004). Implicit coordination is an adjustment behavior driven by deep cognition, which is a behavior spontaneously generated by employees. Explicit coordination is explicit, conscious, and perceptible, while implicit coordination is spontaneous, unconscious, and imperceptible (Cannon-Bowers et al., 1993; Wittenbaum et al., 1996; Espinosa et al., 2004).

We believe that previous studies generally focus on explicit coordination and emphasize the important role of explicit coordination on operational performance, but this only provides a relatively static picture of operation. While their importance is indisputable, explicit coordination mechanisms reveal only one aspect of coordination. Scholars generally believe that “good coordination is almost imperceptible,” that is, implicit coordination is more natural than explicit coordination, and can provide stronger coordination and higher productivity for enterprises. Through the study of cognitive field, we believe that the concept of coordination expands the understanding of the coordination contribution of enterprise employees in the interaction process. Therefore, how to promote the implicit coordination behavior among enterprise employees, lower coordination costs, and process losses, and improve coordination effectiveness has become the focus of modern enterprises (Kachra and White, 2008; Uitdewilligen et al., 2018).

In recent years, organizational research scholars have introduced status research from the field of sociology to the field of management, which is used to explain related management phenomena and problems within and between organizations (Magee and Galinsky, 2008; Piazza and Castellucci, 2014). In contrast to sociologists who apply the concept of status to a wide range of social situations, organizational and management researchers apply it to micro situations, such as competitive environments, markets, organizations, or teams. They tend to seek the positive outcomes that status produces in the field of management (Lee and Jeung, 2018; Lam, 2021).

Since enterprise production is a kind of group behavior, the knowledge mastered by individual employees needs to interact with the knowledge of other employees to produce value. Enterprise employees have different divisions of labor, majors, and knowledge backgrounds. By connecting employees of different majors with corresponding jobs, they can not only play their respective roles, but also promote their own knowledge growth through specialization. However, the cooperation of employees with different backgrounds and professions will bring about conflicts in cognition, relationship, procedures, etc., especially status competition, which is one of the important factors affecting employee cooperation, communication, and coordination.

Status is a very important attribute of an individual in an enterprise (Berger et al., 1972; Anderson et al., 2001; Magee and Galinsky, 2008). Status differences in an enterprise will affect the degree of respect, access to resources, influence on others, and ultimately affect the behavior motivation of individuals. For example, when employees think their status is low, in the need of self-esteem and self-actualization, they will expect to obtain higher status through status-competitive behavior (Berger et al., 1980; Gandz and Murray, 1980; Anderson et al., 2008; Cheng et al., 2013).

Status competition is the effort of employees to change their relative status in the enterprise. Based on different competitive motivations, it can be divided into the prestige-type status competition based on prosocial motivation and the dominant-type status competition based on self-interest motivation (Bendersky and Hays, 2012). Enterprise management practice finds that the motivation of status competition will affect knowledge sharing, experience exchange, and information transfer among employees, especially making the implicit coordination behavior increasingly complicated (Banks et al., 2016; Lee et al., 2017).

When studying the mechanism of employees’ status competition motivation on implicit coordination, knowledge sharing is one of the important perspectives. Cheng et al. (2013) show that prestige-type status competition motivation and dominant-type status competition motivation are positive predictors of an individuals’ interpersonal liking or rejection in the organization. According to social classification theory, in the process of gaining status, employees will socially classify certain characteristics and behaviors of individuals with different motivations and show inconsistent social interaction patterns. Individuals with prestige-type motivation usually show generous and friendly behaviors and will get more praise and preference in interpersonal interactions. To reward the recognition and respect of other employees, they will increase knowledge sharing behaviors. Individuals with dominant-type motivation rarely show interpersonal sensitivity or sympathy for other employees of the enterprise and are easily disgusted and rejected by other employees, thereby reducing their own knowledge sharing behaviors.

It is clear from the above analysis that status differences and status demands can cause individuals to develop prestige-type and dominant-type status competition, and make individuals receive different treatment in the enterprise, such as welcome or exclusion, which affects their decision making and behavioral choices for sharing or hiding knowledge. According to motivated information processing model, individual behavior is not only influenced by their needs and motivations but may also be influenced by the weighting of certain situational factors. Psychological safety is a common feeling of mutual support among employees, which can prompt individuals to change from self-orientation to collective orientation and make more beneficial altruistic behaviors. When employees feel safe, they will have positive learning behavior or innovation behavior (He et al., 2020; Wu and Chen, 2021). Psychological safety will affect employees’ knowledge sharing behavior. When employees perceive a high sense of psychological safety, to consolidate or improve their status in the enterprise, they are more inclined to share knowledge or information, are willing to form better interpersonal relationships and common cognition, and will produce more positive implicit coordination behaviors. Conversely, when the level of psychological safety is low, employees tend to be cautious and conservative, like to hide their knowledge and use it as a political resource to protect their status. Based on this, this research introduces psychological safety as a moderator variable to explore the mechanism of individual competitive motivation, knowledge sharing, and implicit coordination behavior.

In conclusion, status competition motivation often becomes an important factor to stimulate employees’ implicit coordination behavior. Employees with different status competition motivation have different cognition of knowledge sharing, which will have substantial influence on implicit coordination behavior. Therefore, this paper will refer to the logical framework of coordination theory, incorporate the concept of status competition into the field of organizational coordination, systematically study the relationship between status competition motivation and implicit coordination behavior, and test the mediating role of knowledge sharing and the moderating role of psychological safety. The research results can enrich theoretical research in the field of organizational coordination and provide reference for enterprise team management and human resources management in the field of practice.

## Theoretical Basis and Research Hypothesis

According to cognitive theory, motivation is an intrinsic drive that determines behavior. The production of employees’ implicit coordination behavior also has its intrinsic motivation, among which information (knowledge) sharing is one of the main incentives. The motivated information processing model believes that individual decision making is an information processing process (Hinsz et al., 1997). What information is shared, and the quality of information sharing and integration is influenced by two distinct motivations, epistemic motivation, and social motivation. Epistemic motivation is employees’ willingness to make the effort to understand something comprehensively and accurately; social motivation is an individual’s preference for distributing outcomes among themselves and others (De Dreu et al., 2006). Epistemic motivation determines the depth of information processing, while social motivation determines the direction of information processing. The two motivations work together and ultimately determine the quality of decision making (De Dreu et al., 2008).

According to this theory, the internal motivation of employees will affect their behavior; especially the social motivation of employees determines the way and path of information processing to some extent. When employees have prosocial motivation (prestige-type status competition motivation), they are more willing to think about problems and obtain information (knowledge) from the perspective of others, resulting in more willingness to cooperate and share information. Under the influence of prosocial motivation, employees will produce more positive role behaviors (including in-role behavior and out-role behavior), tend to consider themselves and others as a whole to consider joint benefits, and regard cooperation or win-win as the key. When employees have self-interested motivation (dominant-type status competition motivation), they tend to pursue profit maximization, ignore, or even belittle the achievements of others, and regard gaining their own rights as the key to competition. Relevant studies have shown that employees’ intrinsic motivation has a good predictive effect on emotional experience, creative behavior, cooperation persistence, job satisfaction, etc. (Grant and Berry, 2011; Cortina, 2017). Individuals with prosocial motivations can redouble their efforts to maximize joint benefits based on mutual trust. Only on the basis of information processing and knowledge sharing can individuals obtain more comprehensive resources and make more reasonable judgments and decisions, which provide an important foundation for employees to generate implicit coordination behaviors. That is, knowledge sharing, to a certain extent, can determine the communication among employees, which is conducive to promoting the integration of different knowledge and laying a foundation for employees to predict or adjust their behavior.

In addition, since implicit coordination is an effective prediction of employees’ behavior based on each other, it is built on the basis that employees share information or knowledge related to tasks or work with each other and form common cognition. That is, when employees form a consistent cognition of tasks or work goals through knowledge sharing, they can effectively predict the actions and needs of other employees (Rico et al., 2008; Butchibabu et al., 2016). Therefore, implicit coordination requires employees to share more knowledge; sufficient knowledge sharing is the precondition for the formation of implicit coordination. However, due to the different motivation of status competition among employees, there will be differences in knowledge sharing, including sharing willingness, sharing content, sharing methods, and approaches (Estrada et al., 2016). Specifically, employees with prestige-type status competition motivation will take the initiative to help others, share key knowledge, and spread core information to gain respect, which is conducive to the formation of a harmonious working atmosphere in the enterprise and the establishment of mutual trust (Flynn et al., 2006; Cheng et al., 2013). Employees with dominant-type status competition motivation are reluctant to share their knowledge. Due to the difficulty in measuring the value of shared knowledge, the uncertainty of knowledge contribution, and the fear of free-riding effect, employees are reluctant to make efforts, which will lower the intrinsic work motivation and reduce mutual help behavior.

### Status Competition and Implicit Coordination

The obstacle to the implicit coordination of employees is that the coordination contribution cannot be clearly defined, and compensation incentives cannot explicitly compensate employees for the enterprise benefits brought about by implicit coordination (Willer, 2009). However, by providing status resources (such as management ranks), the relationship between status competition behavior and implicit coordination can be established to solve the marginal contribution problem in achieving enterprise task goals.

When individuals realize that their status is low, out of the needs of self-esteem and self-value realization, they will improve their own status level through competitive behavior, that is, the adjustment of status can motivate employees to increase work engagement and create higher personal performance (Nahapiet and Ghoshal, 1998; Kleinbaum and Stuart, 2014). However, the different motivations of individuals determine that they adopt different ways of status competition. Prestige-type status competitors have prosocial motivations and tend to show behaviors such as proactively helping others and proactively sharing information, while dominant-type status competitors have self-interest motivations and are prone to unethical behaviors that harm the interests of others. The former considers status competition to achieve task goals, while the latter considers status competition to achieve individual ends (Loch et al., 2000; Huberman et al., 2004). Therefore, employees with prestige-type status competition motivation will actively anticipate the needs of other employees and adjust their behaviors to gain their own reputation in the enterprise, thereby establishing and consolidating their own status. While employees with dominant-type status competition, motivation prefers not to participate in or even hinder the coordination of others in order to preserve their own resource value and prevent the loss of status.

According to the coordination theory, when dealing with the interdependence of employees, the employees of the prestige-type status competition motivation will be willing to help others and share key information due to their prosocial motivation. It is easy to form a harmonious working atmosphere in the enterprise, and employees have a more trusting relationship and a sense of cooperation and collaboration. When employees are faced with high-risk work tasks, they tend to share risks, resulting in closer interdependence, which is conducive to the generation of implicit coordination behavior (Rico et al., 2019; Espinosa et al., 2021).

Therefore, prestige-type status competition motivation will promote the generation of employees’ implicit coordination behavior. On the contrary, employees with dominant-type status competition motivation are prone to unethical behaviors due to their self-interested motivations and are prone to mutual suppression, malicious exclusion, marginalization, and mutual shirk, resulting in tense interpersonal relationships and fierce competition atmosphere. When employees are faced with high-risk work tasks, they will choose to avoid responsibilities and risks, destroy the interdependence among employees, make management more difficult, and it is difficult to form an effective implicit coordination behavior (Lowry et al., 2013).

Therefore, employees who engage in dominant-type status competition motivation may hinder the coordination behaviors of others or be unwilling to participate in them, ultimately hindering the formation of implicit coordination. However, employees who adopt prestige-type status competition motivation may provide more communication behaviors, which will have a positive impact on implicit coordination. This paper proposes the following hypotheses:

*H1a*: Prestige-type status competition positively affects implicit coordination.
*H1b*: Dominant-type status competition negatively affects implicit coordination.

### The Mediating Role of Knowledge Sharing

The role of knowledge sharing on implicit coordination is reflected in the following aspects. Knowledge sharing helps employees to obtain key information about task work and facilitates the formation of common cognition (Gagné et al., 2019). Knowledge sharing can change employees’ attitudes, perspectives, or perceptions about the path to achieving task goals (Shteynberg and Galinsky, 2011). Knowledge sharing helps to improve mutual understanding among employees and better promote interdependence (Huber and Lewis, 2010; Randolph-seng and Norris, 2011).

The successful experience of many enterprises shows that employees must reach a common understanding in performing tasks and coordinating behaviors, which can be better positioned to anticipate the needs and actions of other employees, thereby increasing work performance (Cannon-Bowers et al., 1993). Knowledge sharing helps employees to develop distributed expertise, form shared and accurate common cognition, and thus enhance individual’s full understanding of others’ behavior, intention, and cognition (Bock et al., 2005; Navimipour and Charband, 2016). Knowledge sharing can also increase the familiarity and trust among employees, increase the frequency of communication and interaction, and more accurately understand the mental models of other employees, thus making it easier to form implicit coordination (Klimoski and Mohammed, 1994; Stenius et al., 2017; Singh et al., 2021).

According to coordination theory, coordination is the process of managing interdependence. When interdependence is high, employees can share and coordinate their task inputs (such as information, knowledge, and other resources) to complete the work smoothly (Alper et al., 1998). Conversely, employees may be split and work individually. Through knowledge sharing, enterprise employees can not only quickly understand what other employees are doing, but also can predict what other employees may do or need (Lewis and Herndon, 2011; Mell et al., 2014; Choi et al., 2020). Based on the altruistic motivations, prestige-type status competitors give help to others, thus forming a high interdependence relationship, which is conducive to promoting the formation of implicit coordination through knowledge sharing. On the contrary, the dominant-type status competitors have a more tense relationship with other employees. The self-interested motivations destroy the interdependence with other employees; easily produce knowledge hiding behaviors, and cause obstacles to implicit coordination (He et al., 2021).

It can be seen that prestige-type status competitors with altruistic motivations have a strong willingness to share knowledge (Bendersky and Hays, 2012) and will gain respect by helping others and sharing key information, which can promote implicit coordination behavior. Dominant-type status competitors with self-interested motivations pay more attention to personal goals and are unwilling to contribute knowledge to achieve goals, which will inhibit implicit coordination. Therefore, this paper proposes the following hypotheses:

*H2a*: Knowledge sharing plays a mediating role in the relationship between prestige-type status competition and implicit coordination.
*H2b*: Knowledge sharing plays a mediating role in the relationship between dominant-type status competition and implicit coordination.

### The Moderating Role of Psychological Safety

At the individual level, psychological safety is a feature that reflects an individual’s internal psychological state and self-perception (Schein and Bennis, 1965). It is a general feeling and common belief that individuals support each other, that is, it is safe to take interpersonal risks in an enterprise. The establishment of this common belief is rooted in mutual trust, mutual respect, and mutual care among employees. It is also rooted in the perception of employees that their self-image, status, and career will not suffer negative consequences when expressing and presenting themselves (Kahn, 1990; He et al., 2020). Employees have a sense of risk sharing in the context of psychological safety, which is a driving force for free, open, candid communication and coordination (Edmondson, 1999; Newman et al., 2017). High psychological safety promotes employees’ willingness to share knowledge, experiences, and practices, and to agree on tasks goals. It also frees employees from concerns about expressing opinions and discussing issues, as well as letting others know about their expected actions. Low psychological safety will make enterprise employees worry that their shared knowledge will be used to harm their own interests (Zeng et al., 2020).

Prestige-type status competitors gain respect from others through unique skills, knowledge, character, etc., thereby increasing their influence over other employees of the enterprise, such as cooperation, mutual trust, and respect for others. Therefore, when the sense of psychological safety is higher, due to the establishment of prestige, the love of self-esteem, and the desire for status, prestige-type status competitors will not be worried and anxious, dare to speak up, have the courage to admit mistakes, and actively share knowledge.

When dominant-type status competitors feel psychologically safe, they are encouraged to express different information and opinions, and to communicate freely without fear of negatively affecting their status (Kahn, 1990). Employees with dominant-type status motivations will change their previous suppression methods and pay more attention to the mistakes and deficiencies of others. That is, by actively speaking, expressing their own opinions, controlling the behavior of other employees, and taking the initiative to improve their status, they increase their confidence in participating in task and goal discussions. Therefore, this paper proposes the following hypotheses:

*H3a*: Psychological safety has a moderating role in the relationship between prestige-type status competition and knowledge sharing, that is, the higher the level of psychological safety, the stronger the positive effect of prestige-type status competition on knowledge sharing.
*H3b*: Psychological safety has a moderating role in the relationship between dominant-type status competition and knowledge sharing, that is, the higher the level of psychological safety, the weaker the negative impact of dominant-type status competition on knowledge sharing.

**Figure 1:**
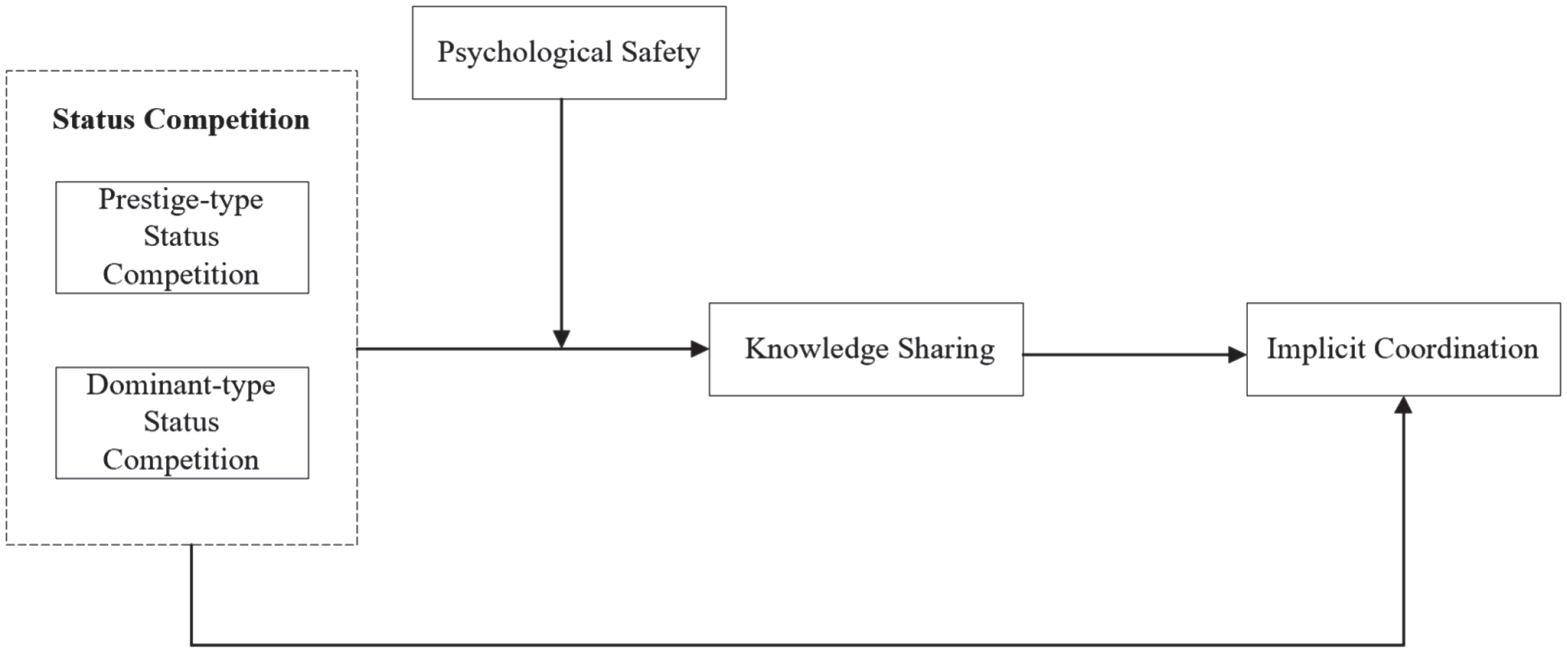
The conceptual mode of this research.

The conceptual mode of this research is shown in Figure 1.

## Research Methods

### Samples and Procedures

The data of this research come from Jiangsu, Shanghai, Beijing, Zhejiang, and other places in China, covering task teams in manufacturing enterprises, service enterprises, high-tech enterprises, and so on. To ensure data quality, questionnaires were filled out by task employees. In the formal research stage, we used professional research companies and important contacts of enterprises and other channels. And we distributed questionnaires through the enterprise WeChat group, Email, APP, and personal contact information in the form of network link. Before filling in the questionnaires, we obtained the consent of the managers of the human resources departments of each enterprise and explained the purpose, process, and confidentiality matters of the survey to the respondents, to ensure that the respondents could fill in the questionnaires according to the real situation.

A total of 500 questionnaires were distributed in this survey, and 420 questionnaires were recovered, with a recovery rate of 84%. After eliminating invalid questionnaires, 367 valid questionnaires were obtained, and the effective recovery rate reached 91.75%. Table 1 shows the organizational characteristics, and Table 2 shows the employee characteristics.

**Table 1:** Characteristics of organizations.

Size (%)		Date of establishment (%)		Industry type (%)		Location (%)	
<50	43.20%	<3	13.60%	Manufacturing	40.90%	Jiangsu	68.2%
51 ~ 500	20.50%	3 ~ 5	11.40%	Service	13.60%	Shanghai	11.4%
501 ~ 1,000	15.90%	6 ~ 10	15.90%	Wholesale	13.60%	Beijing	4.5%
1,001 ~ 5,000	18.20%	>10	59.10%	High-tech	18.20%	Zhejiang	2.3%
> 5,000	18.10%			Others	13.70%	Others	13.6%

**Table 2:** Characteristics of employees.

Gender (%)		Age (%)		Education level (%)		Years in the company (%)		Years in the team (%)	
Male	53.10%	<24	12.50%	High school or below	11.20%	<1	21.80%	<6 months	17.40%
Female	46.90%	25–30	22.10%	Junior college	25.30%	1 ~ 2	28.10%	7–12 months	18.80%
		31–35	39.20%	Bachelor degree	55.90%	3 ~ 5	24.50%	1–3 years	29.70%
		36–40	2.50%	Master	5.50%	6 ~ 10	15.50%	4–6 years	14.20%
		41–45	13.40%	Doctor	2.10%	>10	10.10%	>7 years	19.90%
		>46	10.30%						

### Measurement of Variables

The scales used in this research are all mature scales proposed by previous scholars, and the scales were translated into Chinese according to the procedures of translation and retranslation, to ensure the consistency of the original meaning in different semantic contexts. All measurement items in the questionnaire are based on a five-point Likert scale, where 1 means “completely disagree” and 5 means “completely agree.”

#### Status Competition

Referring to the measurement items of Huberman et al. (2004) and Bendersky and Hays (2012) this paper uses 11 items to measure the status competition behavior and adjust it according to specific situations. The prestige-type status competition includes five items, such as “The rest of the enterprise employees respects me” and “I am often asked for advice and help at work.” The scale’s internal consistency coefficient is 0.633. The dominant-type status competition includes six items, such as “I often try to achieve my goals regardless of what other people think” and “I try to control others and not allow others to control me.” The scale’s internal consistency coefficient is 0.903.

#### Implicit Coordination

This paper uses the implicit coordination measurement scale developed by Rico et al. (2008) and Khan et al. (2010), which includes eight measurement items, such as: “I can anticipate the actions of employees without communication” and “I adapt my approach to achieve the task’ shared goals.” The scale’s internal consistency coefficient is 0.780.

#### Knowledge Sharing

This paper refers to the measurement items of Bock et al. (2005) and uses six items to measure knowledge sharing willingness, including explicit knowledge sharing and implicit knowledge sharing, such as: “I often share my documents and reports with other employees” and “I will share my working methods and models with other employees.” The scale’s internal consistency coefficient is 0.859.

#### Psychological Safety

This paper refers to the psychological safety scale of Edmondson (1999) and adjusts it in combination with specific situations, using five measurement items, including “If I make a mistake at work, others will complain about me” and “In an enterprise, I can ask questions and stick to my opinion.” The scale’s internal consistency coefficient is 0.563.

#### Control Variables

Previous studies suggest that demographic variables, such as gender, age, and education level and factors such as the length of time an individual has joined a company and a team can affect the motivation or behavior of employees’ knowledge sharing and implicit coordination. Most empirical studies on status competition and implicit coordination also use these variables as control variables (Chang et al., 2017; He et al., 2021). Therefore, the control variables considered in this research include gender, age, education level, years in the company, and years in the team.

## Data Analysis and Results

### Descriptive Statistics and Correlation Analysis

The descriptive statistics and correlation analysis results of the main research variables involved in this research are shown in Table 3. It can be seen from Table 3 that prestige-type status competition and knowledge sharing (*r* = 0.299, *p* < 0.01) and implicit coordination behavior (*r* = 0.343, *p* < 0.01) are significantly positively correlated. Knowledge sharing and implicit coordination are also significantly positively correlated (*r* = 0.520, *p* < 0.01). Dominant-type status competition was significantly negatively correlated with knowledge sharing (*r* = −0.293, *p* < 0.01) and significantly negatively correlated with implicit coordination behavior (*r* = −0.184, *p* < 0.01). In addition, there was a significant positive correlation between psychological safety and implicit coordination (*r* = 0.265, *p* < 0.01). All main research variables were roughly moderately correlated, and in the same direction as the previous hypothesis, suitable for further analysis.

**Table 3:** Descriptive statistics and correlations among all variables.

Variables	*M*	*SD*	1	2	3	4	5
1. Prestige-type status competition	3.718	0.727	1				
2. Dominant-type status competition	2.22	0.966	−0.036	1			
3. Knowledge sharing	4.095	0.673	0.299^**^	−0.293^**^	1		
4. Psychological safety	3.837	0.387	0.135^*^	0.080	0.058^**^	1	
5. Implicit coordination	3.600	0.654	0.343^**^	0.520^**^	0.505^**^	0.265^**^	1

*N = 367*.

* *p* < 0.05;

** *p < 0.01*.

### Common Method Biases Test

This paper mainly uses two methods to test the common method deviation: one is to use the Harman single factor test method. And use the principal component analysis method to conduct exploratory factor analysis on all test items, a total of eight factors are separated out. The first factor explains only 25.729% of the total variance, which is much less than 50%, that is, there is no single factor explaining most of the variance. The second is to use confirmatory factor analysis to build a competitive model to test the fitting effect of sample data. The specific analysis results are shown in Table 4. The five-factor model has the best fitting effect with the observed data (χ^2^/df = 2.732, RMSEA = 0.072, CFI = 0.838, TLI = 0.821, SRMR = 0.089).

**Table 4:** Results of the confirmatory factor analysis for the main variables.

	**χ** ^2^	df	**χ**^2^/df	RMSEA	CFI	TLI	SRMR
Single-factor model: WDW + ZDW + KS + PS + IC	2579.702	405	6.370	0.127	0.484	0.445	0.125
Two-factor model: WDW and ZDW + KS + PS + IC	2262.767	404	5.601	0.117	0.559	0.525	0.120
Three-factor model: WDW and ZDW and KS + PS + IC	1464.296	402	3.643	0.087	0.716	0.692	0.103
Four-factor model: WDW and ZDW and KS and PS + IC	1162.926	399	2.915	0.076	0.819	0.802	0.088
Five-factor model: WDW and ZDW and KS and PS and IC	1079.043	395	2.732	0.072	0.838	0.821	0.089

*WDW, prestige-type status competition; ZDW, dominant-type status competition; KS, knowledge sharing; PS, psychological safety; and IC, implicit coordination. The evaluation standard of index goodness of fit is: χ^2^/df < 3.2, RMSEA < 0.08, CFI > 0.76, TLI > 0.74, and SRMR < 0.103. *N* = 367*.

### Hypothesis Testing

For the research hypothesis, firstly, hierarchical regression analysis was used to examine the direct effect of status competition on implicit coordination behavior, the mediating effect of knowledge sharing, and the moderating effect of psychological safety. The specific regression results are shown in Tables 5, 6.

**Table 5:** Regression analysis of prestige-type status competition.

	Knowledge sharing			Implicit coordination			
	M1	M2	M3	M4	M5	M6	M7
Gender	−0.025	−0.001	0.002	0.189	0.235^*^	0.200^*^	0.235^**^
Age	0.046	0.001	−0.006	−0.061	−0.102^*^	−0.076^*^	−0.102^**^
Education level	0.004	0.010	0.027	0.023	0.029	0.024	0.023
Years in the company	−0.130^*^	−0.081^*^	0.006^*^	0.014	0.016	0.075	0.070
Years in the team	0.112	0.060	0.005	0.048	0.027	−0.013	−0.016
Prestige-type status competition		0.217^***^	0.313^***^		0.371^***^		0.227^***^
Knowledge sharing						0.521^***^	0.469^***^
Psychological safety			0.043				
Prestige-type status competition* psychological safety			0.123^**^				
*R* ^2^	0.018	0.104	0.112	0.019	0.146	0.293	0.347
*R*^2^ Change		0.089	0.102		0.127	0.274	0.329
Adjusted *R*^2^	0.004	0.088	0.091	0.004	0.130	0.281	0.333
*F*	1.304	6.479^***^	5.351^***^	1.307	9.344^***^	23.563^***^	24.866^***^

* *p* *< 0.05;*

** *p* *< 0.01;*

*** *p < 0.001*.

**Table 6:** Regression analysis of dominant-type status competition.

	Knowledge sharing			Implicit coordination			
	M1	M8	M9	M4	M10	M11	M12
Gender	−0.025	0.008	0.001	0.189	0.184	0.200^*^	0.177
Age	0.046	0.072	−0.002	−0.061	−0.029	−0.076	−0.058
Education level	0.004	0.028	0.033	0.023	0.030	0.024	0.020
Years in the company	−0.130^*^	−0.122^*^	0.014	0.014	0.019	0.075	0.075
Years in the team	0.112	0.126^*^	0.006	0.048	0.066	−0.013	−0.001
Dominant-type status competition		−0.316^**^	−0.334^***^		−0.186^***^		−0.030
Knowledge sharing						0.521^***^	0.494^***^
psychological safety			0.123^*^				
Dominant-type status competition^*^ psychological safety			0.074^*^				
*R* ^2^	0.018	0.023	0.121	0.019	0.048	0.293	0.277
*R*^2^ Change		0.089	0.113		0.033	0.274	0.261
Adjusted *R*^2^	0.004	0.009	0.101	0.004	0.031	0.281	0.261
*F*	1.304	7.044^***^	5.852^**^	1.307	2.775^*^	23.563^***^	17.840^***^

* *p* *< 0.05;*

** *p* *< 0.01;*

*** *p < 0.001*.

#### Direct Effect Test

After controlling for demographic variables such as gender, age, and education level, it can be seen from Model 5 in Table 5 that prestige-type status competition has a significant positive impact on implicit coordination (*β* = 0.371, *p* < 0.001), H1a is supported. From Model 10 in Table 6, the direct effect of dominant-type status competition on implicit coordination is significantly negative (*β* = −0.186, *p* < 0.001), H1b is supported.

#### Mediating Effect Test

Examining the mediating effect of knowledge sharing between prestige-type status competition and implicit coordination. Based on the direct effect of Model 5 prestige-type status competition on implicit coordination in Table 5, knowledge sharing is further added to the regression equation. The analysis results are shown in Model 7. The results show that the knowledge sharing of employees is significantly positively correlated with their implicit coordination behavior (*β* = 0.469, *p* < 0.001), and the positive effect of prestige-type status competition on the implicit coordination behavior of employees was weakened, but still significant (*β* = 0.227, *p* < 0.001), which means that knowledge sharing plays a mediating role between prestige-type status competition and implicit coordination relationship, H2a is supported.

In addition, knowledge sharing plays a mediating role between dominant-type status competition and implicit coordination. Based on the direct effect of Model 10 dominant-type status competition on implicit coordination in Table 6, knowledge sharing is further added to the regression equation. The analysis results are shown in Model 12. The results show that knowledge sharing of employees is positively correlated with their implicit coordination behavior (*β* = 0.494, *p* < 0.001), and the dominant-type status competition becomes insignificant on the implicit coordination behavior of employees, which means that knowledge sharing plays a mediating role between the dominant-type status competition and the implicit coordination, H2b is supported.

#### Moderating Effect Test

The analysis results of the moderating effect are shown in Model 3. The interaction term of prestige-type status competition and psychological safety has a positive impact on knowledge sharing (*β* = 0.123, *p* < 0.01), that is, the higher the psychological safety level of employees, the stronger the positive effect of prestige-type status competition on knowledge sharing, H3a is supported.

To show the moderating effect of psychological safety on the relationship between prestige-type status competition and knowledge sharing, this paper calculates the difference of the influence of prestige-type status competition on knowledge sharing under different levels of psychological safety, taking the mean value lower than and higher than one standard deviation, respectively. When psychological safety is relatively low, the influence coefficient of prestige-type status competition on knowledge sharing is 0.2534 (*p* < 0.001); while when psychological safety is relatively high, the influence coefficient of prestige-type status competition on knowledge sharing is 0.4097 (*p* < 0.001).

For employees with a higher level of psychological safety, prestige-type status competition has a more obvious role in promoting knowledge sharing. Compared with the low psychological safety situation, the promotion effect of prestige-type status competition on knowledge sharing behavior is strengthened in the high psychological safety situation. That is, psychological safety enhances the positive effect of prestige-type status competition on knowledge sharing and has a positive moderating effect on the relationship between the two.

For the moderating effect of psychological safety on the relationship between dominant-type status competition and knowledge sharing, we first analyze the dominant-type status competition and psychological safety. Then, construct the interaction term of dominant-type status competition and psychological safety, and put it psychological safety into the regression equation of dominant-type status competition on knowledge sharing. The analysis results are shown in Model 9. The interaction term of dominant-type status competition and psychological safety has a significant positive impact on knowledge sharing (*β* = 0.074, *p* < 0.05), indicating that psychological safety significantly affects the relationship between independent variable and dependent variable, H3b is supported. The same method is used to calculate the difference of the influence of dominant-type status competition on knowledge sharing under different psychological safety levels.

When psychological safety is relatively high, the influence coefficient of dominant-type status competition on knowledge sharing is −0.2663 (*p* < 0.001). While when psychological safety is relatively low, the influence coefficient of dominant-type status competition on knowledge sharing is −0.4165 (*p* < 0.001). Compared with low-level psychological safety, high-level psychological safety will weaken the negative impact of dominant-type status competition on knowledge sharing.

## Discussion and Conclusion

### Theoretical Contribution

This paper divides the status competition motivations into prestige-type and dominant-type. Through empirical research, this paper explores the influence mechanism of individual status competition motivation on implicit coordination behavior, analyzes the mediating role of knowledge sharing, and the moderating role of psychological safety between status competition and knowledge sharing. The research results show that employees’ status competition motivation has different effects on implicit coordination behavior. The prestige-type status competition has a positive effect on implicit coordination, while the dominant-type status competition has a negative effect on implicit coordination. The conclusion of the research further verifies the view of Cheng et al. (2013), that is, the motivations of employees for status pursuit mainly include dominant-type status competition motivation and prestige-type status competition motivation. The former regard status as an asset and try to gain dominance over resources and others by seeking status; the latter regard status as a responsibility and gain honor and respect by implementing altruistic behavior. Although employees attach great importance to status resources and their symbols, they also have preferences in specific practice. Enterprise employees with different motivations have different focuses and have different attitudes and behaviors toward implicit coordination.

Knowledge sharing plays a mediating role in the relationship between status competition motivations and implicit coordination behavior. Prestige-type status competition promotes employees’ implicit coordination behavior by positively influencing knowledge sharing, while dominant-type competition weakens employees’ implicit coordination behavior by negatively affecting knowledge sharing. Enterprise employees with different status competition motivations have different views on knowledge sharing, which will affect the common cognition of tasks and group goals and have different degrees of influence on implicit coordination behavior.

Psychological safety moderates the effect of status competition on knowledge sharing. That is, when the degree of psychological safety is higher, the positive effect of prestige-type status competition on knowledge sharing behavior is enhanced, while the negative effect of dominant-type status competition on knowledge sharing behavior is weakened.

This paper has the following theoretical contributions. First, this paper analyzes the characteristics of different status competition behaviors and promotes research in the field of status competition. Status is the master-subordinate relationship that employees are in an enterprise and the level difference between different employees. It is one of the very important attributes of an individual in an enterprise. Status competition is the innate instinct of human beings, in the need of self-esteem and self-realization, employees are eager to obtain a higher reputation and status in the enterprise. The positive and negative effects of status competition largely depend on the motivation of competitors, that is, the motivation and purpose of employees’ use of status. Prestige-type status competition employees have prosocial motivations and see status competition as a signal of personal competence and a means to achieve corporate goals. They pay more attention to honor, prestige, and others’ evaluation of themselves, and are eager to be recognized by others. To maintain and gain dignity and self-esteem, they will show more organizational citizenship behaviors, such as helping others or sharing key information (He et al., 2019). Dominant-type status competition employees have self-interest motivations and see status competition to achieve their goals. They gain more power and rewards behind their status, and often use unethical means to undermine the work performance of others to increase their chances of winning in the competition. By introducing different status competition motivations and analyzing their behavioral characteristics, this research finds different influence mechanisms of status competition motivations on implicit coordination behavior, which further enriches the research in the field of status competition and promotes the cross research in the field of status competition and organization coordination.

Second, this research explores the influence mechanism of implicit coordination behavior from the perspective of individual status competition motivation, which enriches the research in the field of coordination. Coordination of work teams is a general phenomenon that aims to integrate and adjust the actions, knowledge, and goals of interdependent employees to achieve a common goal (Rico et al., 2008). While explicit coordination is explicit, conscious, and perceptible, implicit coordination emphasizes spontaneous, unconscious, and imperceptible coordination. Previous studies on implicit coordination are mostly based on cognitive or knowledge perspectives, focusing on the distribution of enterprise knowledge and expertise, cognitive structure of employees, team situational models, etc., but less attention is paid to the perspective of individual motivation, especially the influence of status, power, structure, etc. (Bunderson and Boumgarden, 2010; Van Der Vegt et al., 2010; Bunderson and Reagans, 2011). In traditional enterprise research, the vast majority of studies default to the assumption that there is no status difference among employees, and employees are completely equal in the process of cooperation. This assumption cannot truly characterize the structural relationships of employees in specific practices. Due to individual differences in abilities, personalities, etc., informal status differences will form among employees (Nembhard and Edmondson, 2006). Status differences affect implicit coordination among employees. Therefore, from the perspective of status competition motivation, we deeply explore the influence path and mechanism on employees’ implicit coordination behavior, which is helpful to enrich the research in coordination field.

Third, this study incorporates psychological safety into the research framework of status competition-knowledge sharing. It expands the boundary conditions of the influencing factors of knowledge sharing and enriches the empirical research on psychological safety as a moderator variable. Psychological safety has always been an important research topic in the field of psychology. It mainly reflects the internal psychological state and self-perception of employees, and has an important impact on learning behavior, suggestion behavior, innovative behavior, work engagement and involvement, and work performance. Based on previous research, this paper further confirms the moderating effect of psychological safety in prestige-type status competition-knowledge sharing and dominant-type status competition-knowledge sharing from the individual level, indicating that psychological safety can provide some situational conditions. Under the condition of high psychological safety, the employees of the enterprise feel the general support and common belief within the enterprise and are more able to actively carry out knowledge sharing activities, which is conducive to the formation of common cognition among employees, and then promotes the formation of implicit coordination behaviors.

### Practical Inspiration

This research has strong practical significance. First, organizational managers need to pay attention to the motivation of status competition among employees and guide them to conduct reasonable status competition. Status competition has some adverse effects on employees. For example, in order to gain recognition from others, employees may reduce their own output through group restrictions; in order to legitimize their own status, employees may increase the capital of status competition through conspiracy and other methods, leading to interpersonal tension and intense competition atmosphere within the enterprise; and communication and negotiation may not be effective means to resolve status conflicts and may have a destructive impact on the growth of enterprises. Therefore, organizational managers need to take measures to intervene and manage employees’ status competition and persuade employees to follow the principle of maximizing organizational interests to participate in status competition through benign interaction. In addition, the status hierarchy in an enterprise is dynamic and unstable, and there is also a winner-take-all effect, which can easily lead to the solidification of status. Therefore, managers should design open and flexible status granting standards according to the situation of enterprise management practice and guide the motivation of status competition. No matter what motivations employees have for status competition, appropriate status granting standards can promote employees to change themselves from self-oriented to team-oriented, to share more knowledge, and then promote the formation of implicit coordination behaviors.

Second, managers should strengthen the fairness management within the enterprise and create an organizational atmosphere of mutual trust, fairness, and smooth communication. Fairness has an important impact on employees. Once corporate employees believe that there is a problem with fairness, unproductive competition will easily occur, resulting in waste of resources and a decline in status incentives. Based on social comparison theory, low-status employees are more sensitive to the perception of fairness in status competition than high-status employees. For low-status employees, they prefer to use prestige-type status competition to gain recognition and respect from other employees (Blader and Chen, 2011). When they perceive higher fairness, they have a stronger willingness to share knowledge and expect to gain more recognition and higher prestige in the enterprise. And when they perceive that the competition for status is unfair, they will hide their knowledge to preserve their status. Conversely, for high-status employees, when they perceive fairness, they hide their knowledge to avoid low-status employees posing a threat to their own status. Currently, they tend to adopt the dominant-type status competition (Anderson et al., 2020). Therefore, enterprises need to pay attention to the motivation of employees’ status pursuit, formulate corresponding measures to maintain the fairness in the status adjustment mechanism, and give full play to the role of status incentives (Witkower et al., 2020).

Third, managers should maintain status stability and improve the psychological safety of employees. Status has the characteristics of stability and self-sustainment (Bothner et al., 2011). Employees’ perceptions of status stability will affect the behavioral choice of hiding or sharing knowledge. For example, for high-status employees, if they think their status is stable, they will perceive a higher sense of psychological safety (Kahn, 1990) and have more autonomy. To maintain their status, they will choose to actively share knowledge to improve the overall level of implicit coordination. For low-status employees, due to their low level of psychological safety, they will be more conservative in their actions and have weaker motivation to improve their status. They are more inclined to hide their knowledge and use it as a political resource to preserve their status in the enterprise (Pai and Bendersky, 2019; Bendersky and Brockner, 2020). When the status of enterprise employees’ changes, the motivation for status competition will also change. For example, low-status employees often gain status through prestige-type status competition behavior, while to maintain their original status, high-status employees often take dominant-type status competition behavior. Therefore, enterprises need to maintain the stability of their internal status and enhance the psychological safety of employees, to effectively stimulate employees’ knowledge sharing and implicit coordination behavior.

### Limitations and Future Research

This research still has the following limitations, which needs to be further improved in the follow-up research. First, in terms of research design, this research mainly uses empirical research to verify the theoretical model, and the research results are also discussed based on cross-sectional data. Future research can provide more convincing evidence for the research hypothesis through vertical research and distribute the questionnaires at different time points to explore the changes in the implicit coordination behavior of employees at different time points. Secondly, this paper mainly discusses the relationship between employees’ status competition motivation and implicit coordination behavior from the individual level but does not clearly distinguish leaders and employees. Team leaders with different management styles may have different status competition motivations. In the future, the two types of personnel can be distinguished for in-depth research. Finally, some potential research directions are worth continuing to explore, such as further subdivision of status competition or consideration of other mediating variables to better understand the operating mechanism. Compared with prestige and dominance, existing studies have begun to focus on the relationship between complaisant, coercive (Ketterman and Maner, 2021), and status competition, and the role of workplace exclusion and cognitive trust in it.

## Data Availability Statement

The raw data supporting the conclusions of this article will be made available by the authors, without undue reservation.

## Ethics Statement

Ethical review and approval was not required for the study on human participants in accordance with the local legislation and institutional requirements. Written informed consent for participation was not required for this study in accordance with the national legislation and the institutional requirements.

## Author Contributions

JX designed the research and completed the manuscript. YX and JW designed the research with JX and proposed the discussion. YP revised and checked the whole manuscript in the revision process. All authors contributed to the design and conceptualization of the manuscript, as well as to review and edit the manuscript. All authors contributed to the article and approved the submitted version.

## Funding

This research project was supported by National Natural Science Foundation of China (nos. 71772088, 71702076, and 71602084); Social Science Foundation of Jiangsu Province (no. 20GLB007); and Postgraduate Research & Practice Innovation Program of Jiangsu Province.

## Conflict of Interest

The authors declare that the research was conducted in the absence of any commercial or financial relationships that could be construed as a potential conflict of interest.

## Publisher’s Note

All claims expressed in this article are solely those of the authors and do not necessarily represent those of their affiliated organizations, or those of the publisher, the editors and the reviewers. Any product that may be evaluated in this article, or claim that may be made by its manufacturer, is not guaranteed or endorsed by the publisher.

## References

[ref1] Alper S. Tjosvold D. Law K. S. (1998). Interdependence and controversy in group decision making: antecedents to effective self-managing teams. Organ. Behav. Hum. Decis. Process. 74, 33–52. doi: 10.1006/obhd.1998.2748, PMID: 9719650

[ref2] Anderson C. Ames D. R. Gosling S. D. (2008). Punishing hubris: the perils of overestimating one’s status in a group. Personal. Soc. Psychol. Bull. 34, 90–101. doi: 10.1177/0146167207307489, PMID: 18162658

[ref3] Anderson C. Hildreth J. A. D. Sharps D. L. (2020). The possession of high status strengthens the status motive. Personal. Soc. Psychol. Bull. 46, 1712–1723. doi: 10.1177/0146167220937544, PMID: 32660350

[ref4] Anderson C. John O. P. Keltner D. Kring A. M. (2001). Who attains social status? Effects of personality and physical attractiveness in social groups. J. Pers. Soc. Psychol. 81, 116–132. doi: 10.1037/0022-3514.81.1.116, PMID: 11474718

[ref5] Banks G. C. Pollack J. M. Seers A. (2016). Team coordination and organizational routines: bottoms up—and top down. Manag. Decis. 54, 1059–1072. doi: 10.1108/MD-07-2014-0442

[ref6] Bendersky C. Brockner J. (2020). Mistreatment from peers can reduce the effects of respectful treatment from bosses, and respectful peers can offset mistreatment from bosses. J. Organ. Behav. 41, 722–736. doi: 10.1002/job.2441

[ref7] Bendersky C. Hays N. A. (2012). Status conflict in groups. Organ. Sci. 23, 323–340. doi: 10.1287/orsc.1110.0734, PMID: 35421383

[ref8] Berger J. Cohen B. P. Zelditch M. (1972). Status characteristics and social interaction. Am. Sociol. Rev. 37, 241–255. doi: 10.2307/2093465, PMID: 35358057

[ref9] Berger J. Rosenholtz S. J. Zelditch M. (1980). Status organizing processes. Annu. Rev. Sociol. 6, 479–508. doi: 10.1146/annurev.so.06.080180.002403

[ref10] Blader S. L. Chen Y. R. (2011). What influences how higher-status people respond to lower-status other? Effects of procedural fairness, outcome favorability, and concerns about status. Organ. Sci. 22, 1040–1060. doi: 10.1287/orsc.1100.0558

[ref11] Blickensderfer E. L. Reynolds R. Salas E. Cannon-Bowers J. A. (2010). Shared expectations and implicit coordination in tennis doubles teams. J. Appl. Sport Psychol. 22, 486–499. doi: 10.1080/10413200.2010.507497

[ref12] Bock G. Zmud R. W. Kim Y. Lee J. (2005). Behavioral intention formation in knowledge sharing: examining the roles of extrinsic motivators, social-psychological factors, and organizational climate. MIS Q. 29, 87–111. doi: 10.2307/25148669

[ref13] Bothner M. S. Kim Y. Smith E. B. (2011). How does status affect performance? Status as an asset vs. status as a liability in the PGA and NASCAR. Organ. Sci. 23, 416–433. doi: 10.1287/orsc.1110.0679

[ref14] Bunderson J. S. Boumgarden P. (2010). Structure and learning in self-managed teams: why “bureaucratic” teams can be better learners. Organ. Sci. 21, 609–624. doi: 10.1287/orsc.1090.0483

[ref15] Bunderson J. S. Reagans R. E. (2011). Power, status, and learning in organizations. Organ. Sci. 22, 1182–1194. doi: 10.1287/orsc.1100.0590, PMID: 35173058

[ref16] Butchibabu A. Sparano-Huiban C. Sonenberg L. Shah J. (2016). Implicit coordination strategies for effective team communication. Hum. Factors 58, 595–610. doi: 10.1177/0018720816639712, PMID: 27113991

[ref17] Cannon-Bowers J. A. Salas E. Converse S. (1993). “Shared mental models in expert team decision making,” in Individual and Group Decision Making: Current Issues. ed. Castellan N. J. (Hillsdale, NJ: Erlbaum), 221–246.

[ref18] Chang H. Lin C. Chen C. Ho Y. (2017). Explicit and implicit team coordination: development of a multidimensional scale. Soc. Behav. Personal. 45, 915–929. doi: 10.2224/sbp.5893

[ref19] Cheng J. T. Tracy J. L. Foulsham T. Kingstone A. Henrich J. (2013). Two ways to the top: evidence that dominance and prestige are distinct yet viable avenues to social rank and influence. J. Pers. Soc. Psychol. 104, 103–125. doi: 10.1037/a0030398, PMID: 23163747

[ref20] Choi G. Nam C. Kim S. Jung H. J. Lee C. H. (2020). Where does knowledge-sharing motivation come from? The case of third-party developer in mobile platforms. J. Knowl. Manag. 24, 1681–1704. doi: 10.1108/JKM-08-2019-0449

[ref21] Colman A. M. Gold N. (2018). Team reasoning: solving the puzzle of coordination. Psychon. Bull. Rev. 25, 1770–1783. doi: 10.3758/s13423-017-1399-0, PMID: 29101730

[ref22] Cortina M. D. (2017). Adaptive flexibility, cooperation, and prosocial motivations: the emotional foundations of becoming human. Psychoanal. Inq. 37, 436–454. doi: 10.1080/07351690.2017.1362920

[ref23] De Dreu C. K. W. Beersma B. Stroebe K. Euwema M. C. (2006). Motivated information processing, strategic choice, and the quality of negotiated agreement. J. Pers. Soc. Psychol. 90, 927–943. doi: 10.1037/0022-3514.90.6.927, PMID: 16784343

[ref24] De Dreu C. K. W. Nijstad B. A. van Knippenberg D. (2008). Motivated information processing in group judgment and decision making. Personal. Soc. Psychol. Rev. 12, 22–49. doi: 10.1177/1088868307304092, PMID: 18453471

[ref25] Edmondson A. C. (1999). Psychological safety and learning behavior in work teams. Adm. Sci. Q. 44, 350–383. doi: 10.2307/2666999, PMID: 32448331

[ref26] Espinosa J. A. Armour F. Boh W. F. (2021). Implicit coordination and Enterprise architecting effectiveness. IEEE Trans. Eng. Manag. 99, 1–17. doi: 10.1109/tem.2021.3109219

[ref27] Espinosa J. A. Lerch F. J. Kraut R. E. (2004). “Explicit versus implicit coordination mechanisms and task dependencies: one size does not fit all,” in Team Cognition: Understanding the Factors That Drive Process and Performance. eds. Salas E. Fiore S. M. (Washington, DC: American Psychological Association), 107–129.

[ref28] Estrada I. Faems D. Faria P. D. (2016). Coopetition and product innovation performance: The role of internal knowledge sharing mechanisms and formal knowledge protection mechanisms. Ind. Mark. Manag. 53, 56–65. doi: 10.1016/j.indmarman.2015.11.013

[ref29] Fisher D. M. Bell S. T. Dierdorff E. C. Belohlav J. A. (2012). Facet personality and surface-level diversity as team mental model antecedents: implications for implicit coordination. J. Appl. Psychol. 97, 825–841. doi: 10.1037/a0027851, PMID: 22468847

[ref30] Flynn F. J. Reagans R. E. Amanatullah E. T. Ames D. R. (2006). Helping one's way to the top: self-monitors achieve status by helping others and knowing who helps whom. J. Appl. Soc. Psychol. 91, 1123–1137. doi: 10.1037/0022-3514.91.6.1123 17144769

[ref31] Gagné M. Tian A. W. Soo C. W. Zhang B. Ho K. S. Hosszu K. (2019). Different motivations for knowledge sharing and hiding: the role of motivating work design. J. Organ. Behav. 40, 783–799. doi: 10.1002/job.2364

[ref32] Gandz J. Murray V. V. (1980). The experience of workplace politics. Acad. Manag. J. 23, 237–251. doi: 10.2307/255429

[ref33] Grant A. M. Berry J. W. (2011). The necessity of others is the mother of invention: intrinsic and prosocial motivations, perspective taking, and creativity. Acad. Manag. J. 54, 73–96. doi: 10.5465/amj.2011.59215085

[ref34] He P. Jiang C. Xu Z. Shen C. (2021). Knowledge hiding: current research status and future research directions. Front. Psychol. 12:748237. doi: 10.3389/fpsyg.2021.748237, PMID: 34777143 PMC8586422

[ref35] He P. Peng Z. Zhao H. Estay C. (2019). How and when compulsory citizenship behavior leads to employee silence? A moderated mediation model based on moral disengagement and supervisor-subordinate guanxi views. J. Bus. Ethics 155, 259–274. doi: 10.1007/s10551-017-3550-2

[ref36] He P. Sun R. Zhao H. Zheng L. Shen C. (2020). Linking work-related and non-work-related supervisor subordinate relationships to knowledge hiding: a psychological safety lens. Asian Bus. Manag. doi: 10.1057/s41291-020-00137-9 [Epub ahead of print].

[ref37] Hinsz V. Tindale R. S. Vollrath D. A. (1997). The emerging conceptualization of groups as information processors. Psychol. Bull. 121, 43–64. doi: 10.1037/0033-2909.121.1.43, PMID: 9000891

[ref38] Huber G. P. Lewis K. (2010). Cross-understanding: implications for group cognition and performance. Acad. Manag. Rev. 35, 6–26. doi: 10.5465/amr.35.1.zok6

[ref39] Huberman B. A. Loch C. H. ÖNçüler A. (2004). Status as a valued resource. Soc. Psychol. Q. 67, 103–114. doi: 10.1177/019027250406700109, PMID: 35136854

[ref40] Kachra A. White R. E. (2008). Know-how transfer: the role of social, economic/competitive, and firm boundary factors. Strateg. Manag. J. 29, 425–445. doi: 10.1002/smj.668

[ref41] Kahn W. A. (1990). Psychological conditions of personal engagement and disengagement at work. Acad. Manag. J. 33, 692–724. doi: 10.5465/256287

[ref42] Ketterman A. B. Maner J. K. (2021). Complaisant or coercive? The role of dominance and prestige in social influence. Pers. Individ. Differ. 177:110814. doi: 10.1016/j.paid.2021.110814, PMID: 33817458

[ref43] Khan M. M. Lodhi S. A. Makki M. (2010). Moderating role of team working environment between team implicit coordination and performance. South African J. Bus. Manag. 4, 2743–2752. doi: 10.5897/ajbm.9000418

[ref44] Kleinbaum A. M. Stuart T. E. (2014). Network responsiveness: the social structural microfoundations of dynamic capabilities. Acad. Manag. Perspect. 28, 353–367. doi: 10.5465/amp.2013.0096

[ref45] Klimoski R. Mohammed S. (1994). Team mental model: construct or metaphor? J. Manag. 20, 403–437. doi: 10.1016/0149-2063(94)90021-3

[ref46] Lam W. M. (2021). A theory of status and coordination in organizations. Oxf. Econ. Pap. 73, 837–855. doi: 10.1093/oep/gpaa025, PMID: 34840395

[ref47] Lee J. W. Jeung C. (2018). Employee status and the consequences of perceived organizational support. J. Pers. Psychol. 17, 75–82. doi: 10.1027/1866-5888/a000198

[ref48] Lee J. Min J. Lee H. (2017). Setting a knowledge boundary across teams: knowledge protection regulation for inter-team coordination and team performance. J. Knowl. Manag. 21, 254–274. doi: 10.1108/JKM-04-2016-0163

[ref49] Lewis K. Herndon B. (2011). Transactive memory systems: current issues and future research directions. Organ. Sci. 22, 1254–1265. doi: 10.1287/orsc.1110.0647

[ref50] Loch C. H. Stout S. K. Huberman B. A. (2000). Status competition and performance in work groups. J. Econ. Behav. Organ. 43, 35–55. doi: 10.1016/S0167-2681(00)00107-4, PMID: 29425972

[ref51] Lowry P. B. Roberts T. L. Romano N. C. (2013). What signal is your inspection team sending to each other? Using a shared collaborative interface to improve shared cognition and implicit coordination in error-detection teams. Int. J. Hum. Comput. Stud. 71, 455–474. doi: 10.1016/j.ijhcs.2012.11.004

[ref52] Magee J. C. Galinsky A. D. (2008). Social hierarchy: the self-reinforcing nature of power and status. Acad. Manag. Ann. 2, 351–398. doi: 10.5465/19416520802211628

[ref53] Malone T. W. Crowston K. (1992). Toward an Interdisciplinary Theory of Coordination, Center for Coordination Science. Cambridge, MA: MIT Press.

[ref54] Mell J. N. Knippenberg D. V. Ginkel W. P. (2014). The catalyst effect: the impact of transactive memory system structure on team performance. Acad. Manag. J. 57, 1154–1173. doi: 10.5465/amj.2012.0589

[ref55] Nahapiet J. Ghoshal S. (1998). Social capital, intellectual capital, and the organizational advantage. Acad. Manag. Rev. 23, 242–266. doi: 10.5465/amr.1998.533225, PMID: 32614865

[ref56] Navimipour N. J. Charband Y. (2016). Knowledge sharing mechanisms and techniques in project teams: literature review, classification, and current trends. Comput. Hum. Behav. 62, 730–742. doi: 10.1016/j.chb.2016.05.003

[ref57] Nembhard M. I. Edmondson A. A. (2006). Making it safe: the effects of leader inclusiveness and professional status on psychological safety and improvement efforts in health care teams. J. Organ. Behav. 27, 941–966. doi: 10.1002/job.413

[ref58] Newman A. Donohue R. Eva N. (2017). Psychological safety: a systematic review of the literature. Hum. Res. Manage. R. 27, 521–535. doi: 10.1016/j.hrmr.2017.01.001, PMID: 35365039

[ref59] Pai J. Bendersky C. (2019). Team status conflict. Curr. Opin. Psychol. 33, 38–41. doi: 10.1016/j.copsyc.2019.07.001 31365895

[ref60] Piazza A. Castellucci F. (2014). Status in organization and management theory. J. Manag. 40, 287–315. doi: 10.1177/0149206313498904

[ref61] Randolph-seng B. Norris J. I. (2011). Cross-understanding in groups: how to “cross Over” Without “dying”. Acad. Manag. Rev. 36, 420–422. doi: 10.5465/amr.2010.0177

[ref62] Rico R. Gibson C. B. Sánchez-Manzanares M. Clark M. A. (2019). Building team effectiveness through adaptation: team knowledge and implicit and explicit coordination. Organ. Psychol. Rev. 9, 71–98. doi: 10.1177/2041386619869972

[ref63] Rico R. Sánchez-Manzanares M. Gil F. Gibson C. (2008). Team implicit coordination processes: a team knowledge–based approach. Acad. Manag. Rev. 33, 163–184. doi: 10.5465/amr.2008.27751276

[ref64] Schein E. H. Bennis W. G. (1965). Personal and Organizational Change Through Group Methods: The Laboratory Approach. New York: Wiley.

[ref65] Shteynberg G. Galinsky A. D. (2011). Implicit coordination: sharing goals with similar others intensifies goal pursuit. J. Exp. Soc. Psychol. 47, 1291–1294. doi: 10.1016/j.jesp.2011.04.012

[ref66] Singh S. K. Mazzucchelli A. Vessal S. R. Solidoro A. (2021). Knowledge-based HRM practices and innovation performance: role of social capital and knowledge sharing. J. Int. Manag. 27:100830. doi: 10.1016/j.intman.2021.100830

[ref67] Stefanini A. Aloini D. Gloor P. (2020). Silence is golden: the role of team coordination in health operations. Int. J. Oper. Prod. Manag. 40, 1421–1447. doi: 10.1108/IJOPM-12-2019-0792

[ref68] Stenius M. Haukkala A. Hankonen N. Ravaja N. (2017). What motivates experts to share? A prospective test of the model of knowledge-sharing motivation. Hum. Res. Manag. 56, 871–885. doi: 10.1002/hrm.21804

[ref69] Stout R. J. Cannon-Bowers J. A. Salas E. Milanovich D. M. (1999). Planning, shared mental models, and coordinated performance: an empirical link is established. Hum. Factors 41, 61–71. doi: 10.1518/001872099779577273

[ref70] Uitdewilligen S. Rico R. Waller M. J. (2018). Fluid and stable: dynamics of team action patterns and adaptive outcomes. J. Organ. Behav. 39, 1113–1128. doi: 10.1002/job.2267

[ref71] Van Der Vegt G. S. De Jong S. B. Bunderson J. S. Molleman E. (2010). Power asymmetry and learning in teams: the moderating role of performance feedback. Organ. Sci. 21, 347–361. doi: 10.1287/orsc.1090.0452

[ref72] Willer R. (2009). Groups reward individual sacrifice: The status solution to the collective action problem. Am. Sociol. Rev. 74, 23–43. doi: 10.1177/000312240907400102

[ref73] Witkower Z. Tracy J. L. Cheng J. T. Henrich J. (2020). Two signals of social rank: prestige and dominance are associated with distinct nonverbal displays. J. Pers. Soc. Psychol. 118, 89–120. doi: 10.1037/pspi0000181, PMID: 31021104

[ref74] Wittenbaum G. M. Stasser G. Merry C. J. (1996). Tacit coordination in anticipation of small group task completion. J. Exp. Soc. Psychol. 32, 129–152. doi: 10.1006/jesp.1996.0006

[ref75] Wu Y. J. Chen J. C. (2021). Stimulating innovation with an innovative curriculum: a curriculum design for a course on new product development. Int. J. Manag. Educ. Oxf. 19:100561. doi: 10.1016/j.ijme.2021.100561

[ref76] Zeng H. Zhao L. Zhao Y. (2020). Inclusive leadership and taking-charge behavior: roles of psychological safety and thriving at work. Front. Psychol. 11:62. doi: 10.3389/fpsyg.2020.00062, PMID: 32153448 PMC7044413

